# Effect of methyl salicylate (MeSA), an elicitor on growth, physiology and pathology of resistant and susceptible rice varieties

**DOI:** 10.1038/srep34498

**Published:** 2016-10-11

**Authors:** Kandaswamy Kalaivani, Marimuthu Maruthi Kalaiselvi, Sengottayan Senthil-Nathan

**Affiliations:** 1 Post Graduate and Research Centre, Department of Zoology, Sri Parasakthi College for Women, Courtrallam-627 802, Tirunelveli, Tamil Nadu, India; 2 Division of Biopesticides and Environmental Toxicology, Sri Paramakalyani Centre for Excellence in Environmental Sciences, Manonmaniam Sundaranar University, Alwarkurichi – 627 412, Tirunelveli, Tamil Nadu, India

## Abstract

Methyl salicylate (MeSA) is a volatile organic compound synthesized from salicylic acid (SA) a plant hormone that helps to fight against plant disease. Seed treatment with MeSA, is an encouraging method to the seed industry to produce more growth and yield. The aim of our study is to find out the growth, development and disease tolerance of rice seed treated with different concentrations of MeSA. Also the seed treatments were studied to determine whether they directly influenced seedling emergence and growth in rice (*Oryza sativa* L) cultivars ‘IR 20, IR 50, IR 64, ASD 16, ASD 19 and ADT 46’ under greenhouse condition. MeSA seed treatments at 25, 50, 75 and 100 mg/L significantly increased seedling emergence. Effects were stronger in IR 50, and IR 64 and the effects were dose dependent, although the relationship between dose and effect was not always linear. MeSA seed treated rice plant against bacterial blight were analyzed. Bacterial blight was more effectively controlled by the seed treated with 100 mg/L than others. These results suggest that seed treatment with MeSA alters plant physiology in ways that may be useful for crop production as well as protection.

Plants are one of the basic staple food, for all forms of life. We depend on plants for food and medicine, plant derivatives, regulation of the hydrologic cycle. Plants are essential to the balance of nature^1^. For more than half of the world’s population “Rice is life”. Rice is an economic crop for the farmers and major food staple for the world. Rice provides the main source of food energy for at least one third of the world’s population^2^,^3^,^4^. Rice is an essential part of the daily meal in Southern and Eastern parts of India and Asia. However most the rice plant are affected by the Bacterial blight caused by *Xanthomonas oryzae* pv. *oryzae (Xoo*) and it is one of the most important bacterial diseases of rice. Heavy yield losses due to *X. oryzae* was reported during last one decade^3^,^5^,^6^.

Seed germination is one of the early and vital stages in the plant life cycle. Improved germination of seeds is one process which can improve the economic success of crop by ultimately enhancing the yield. The process of seed germination may be regulated by external factors, including temperature, light, soil moisture and salinity, metal or mineral composition^7^,^8^.

Treatments of seed include applications of chemical agents, biological substances, or physical processes, to increase agricultural production. The seed treatment helps to protect seedlings from insects and disease which pose devastating consequences to crop production during critical growth stages. The application of treatments to seeds promotes stronger and healthier roots, leading to uniform seedling emergence, with improved crop performance^9^,^10^.

Seed priming is a method of pre-sowing treatments that exposes seeds to a certain solutions that allows partial hydration until the germination process begins^11^,^12^,^13^.Various seed priming techniques have been reported to enhance germination and allow for more uniform seed emergence, increasing nutrient reserves through increased physiological activities and increased yield in the crop^14^.

Various common methods utilized for priming seeds includes: hydro priming, (soaking in water); osmopriming (soaking seed in osmotic solutions); thermopriming (treatment of seed with low or high temperatures); solid matrix priming (treatment of seed with solid matrices); and bio priming (hydration using biological compounds)^15^. Halo priming (soaking seed in salt solutions), and priming with plant growth hormones are also performed along with thermo priming, and on-farm priming^16^.

The mechanism of rice seed priming techniques which uses plant growth regulators may enhance rice performance. Plant hormones or bio-stimulants are also known to improve plant population and increase productivity. Presoaking seeds with optimal concentrations of plant growth hormones, such as auxin (IAA), gibberellins (GA), abscisic acid, ethylene (ET), Salicylic acid (SA) and Jasmonic acid (JA) have been shown to benefit germination performance and yield of some crop species growth under both normal and stress conditions^17^,^18^,^19^. The effects of seed priming by soaking seeds of sorghum, rice, or wheat in water, are reported to enhance germination rates. Some strategies for more vigorous plants report improved seedling emergence under induced stress conditions^20^,^21^.

SA is a natural phenolic compound that plays a role in the regulation of physiological and biochemical processes. SA is chemically known as 2-hydroxy benzoic acid is a member of phenolic compounds, containing of an aromatic ring bearing a hydroxyl group which is synthesized by the plant^22^. These hydroxyl groups are involved in lignin biosynthesis; others serve as allelopathic compounds, regulate plant responses to abiotic stress and disease resistance by inducing antimicrobial defense compounds termed phytoalexins or by signaling defense activation^23^,^24^,^25^,^26^.

Exogenous application of SA act as potential antioxidant capacity inducers, also increase the levels of hydrogen peroxide in plants. These pathways also regulate many physiological processes in plants, such as increased germination rate, cell growth, stomatal opening, photosynthesis and ion uptake^27^. Methyl salicylate (MeSA) is a volatile organic compound which is synthesized from SA. MeSA is associated with induced resistance plant defense which has been used in effective disease control methods^28^,^29^,^30^.

Seed treatment with MeSA can be used to enhance rice seed germination and seedling growth. We studied the performance of rice seed priming with different concentrations of MeSA (0, 25, 50, 75 and 100 mg/L). Variables assessed include: germination rate, growth of root and shoot heights (cm), biomass of fresh and dry weights of the shoots and roots (mg) disease incidence, mean lesion lengths, disease index and disease incidence of different rice varieties under greenhouse conditions.

## Results

### Germination responses after seed treatment with MeSA on rice seed in greenhouse condition

**Figure 1:**
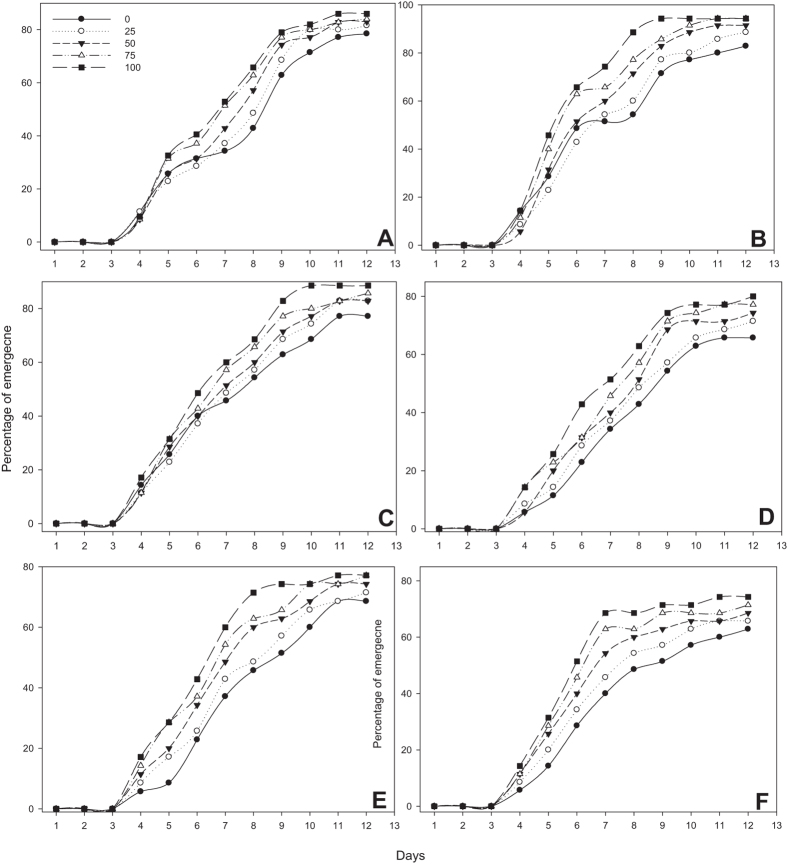
Percentage of emergence of rice varieties treatment with MeSA across 12 days post treatment.(A-IR 20; B-IR 50; C- IR 64; D-ASD 16; E-ASD 19; F-ADT 46).

**Figure 2:**
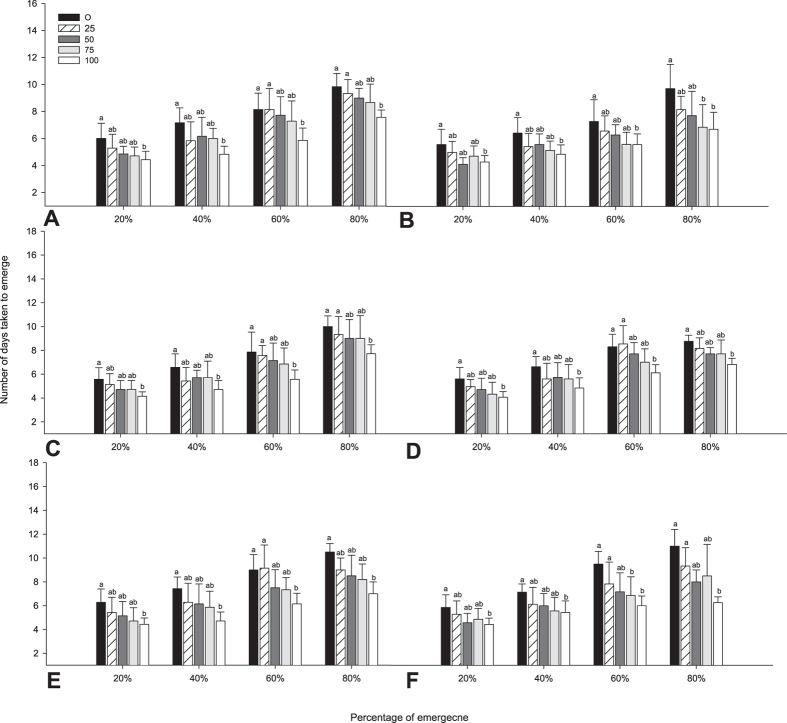
Days until 20%,40%, 60% and 80% emergence of rice varieties IR 20 (**A**), IR 50 (**B**), IR 64 (**C**), ASD 16 (**D**), ASD 19 (**E**), ADT 46 (**F**) under greenhouse condition after seed treatment with Me SA. Mean (±SEM) followed by the same letter in an individual experiment indicate no significant difference (P < 0.05) in a Tukey’s test (Treatment concentration in mg/L).

The seed pretreatment of different concentration of MeSA (i.e. 0, 25, 50, 75 and 100 mg/L) had significant (P < 0.05) effects on the final germination percentage and germination rates (Figs 1 and 2). Treated seeds with MeSA had significantly higher percentage of germination than those in control. Treatment of ‘IR 20, IR 50, IR 64, ASD 16, ASD 19 and ADT 46’ rice varieties with MeSA increased the rate of seedling emergence (Fig. 1A–F). Emergence of rice varieties were more pronounced in all MeSA treatment with 100 mg/L concentration being statistically different with all other treatments. For example 80% germination was observed within 8 days in IR 20 rice varieties after seed treated with 100 mg/L. All the rice varieties treated with MeSA, had greater emergence values compared with control rice plant. In ‘IR 20’ 20% emergence, 40% emergence, 60% emergence and 80% emergence occurred earlier in MeSA treated plants than in control plants (20%-*F*_4,30_ = 3.08; *P* < 0.0031; 40%- *F*_4,30_ = 2.75; *P* < 0.046, 60%- *F*_4,30_ = 3.56; *P* < 0.08 and 80%- *F*_4,25_ = 5.10; *P* < 0.004) (Fig. 2A) there was a trend towards faster emergence with increased concentration seed treatment rates. The same developments were observed in rice variety ‘IR 50’ seedling emergence (20%-*F*_4,30_ = 2.62; *P* < 0.055; 40%- *F*_4,30_ = 3.24; *P* < 0.025, 60%- *F*_4,30_ = 2.65; *P* < 0.053 and 80%- *F*_4,29_ = 4.24; *P* < 0.008) (Fig. 2B).

Germination percentage was influenced by MeSA seed treatment, but influence was more pronounced in 100 mg/L than other treatments. Control seeds emerged at lower than 20% at 4 days. However most of the treated rice seeds germinated at 20%, except (IR 20 and ASD 19), at 4 days, being statistically significant. The same observation was noticed in rice varieties ‘IR 64’ (20%-*F*_4,30_ = 3.28; *P* < 0.024; 40%- *F*_4,30_ = 2.48; *P* < 0.065, 60%- *F*_4,30_ = 2.84; *P* < 0.041 and 80%- *F*_4,26_ = 2.33; *P* < 0.082) (Fig. 2C), ‘ASD 16’ (20%-*F*_4,30_ = 3.05; *P* < 0.032; 40%- *F*_4,30_ = 2.31; *P* < 0.081, 60%- *F*_4,28_ = 5.49; *P* < 0.002 and 80%- *F*_4,13_ = 2.95; *P* < 0.061) (Fig. 2D).

The germination rate of seeds was increased after treatment with MeSA in resistant rice varieties ASD 19 (20%-*F*_4,30_ = 3.06; *P* < 0.031; 40%- *F*_4,30_ = 3.80; *P* < 0.013, 60%- *F*_4,28_ = 5.61; *P* < 0.002 and 80%- *F*_4,13_ = 3.19; *P* < 0.049) (Fig. 2E) and ADT 46 (20%-*F*_4,30_ = 2.88; *P* < 0.040; 40%- *F*_4,30_ = 2.78; *P* < 0.045, 60%- *F*_4,27_ = 5.46; *P* < 0.002 and 80%- *F*_4,11_ = 3.17; *P* < 0.058) (Fig. 2F). Compared with control, the seeds showed greater germination rates when treated with MeSA. The germination rates were significantly greater when treated with MeSA at 100 mg/L.

**Figure 3:**
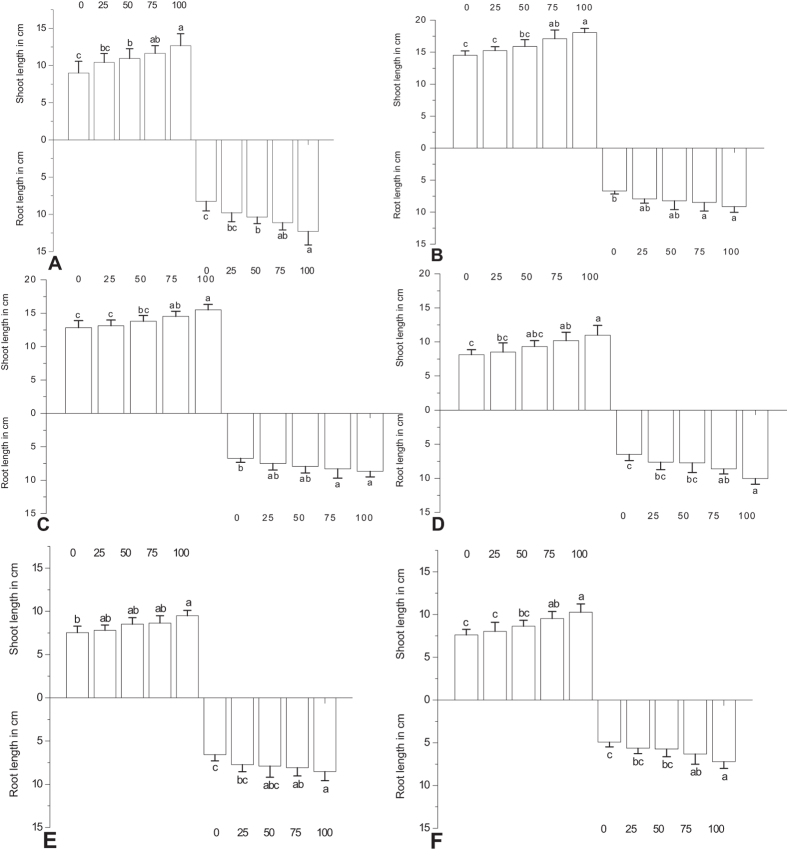
Root and shoot length in cm of rice varieties 22 days post treatment with MeSA (A-IR 20; B-IR 50; C- IR 64; D-ASD 16; E-ASD 19; F-ADT 46). Mean (±SEM) followed by the same letter in an individual experiment indicate no significant difference (P < 0.05) in a Tukey’s test (Treatment concentration in mg/L).

Seed-priming treatments with MeSA significantly enhanced the seedling growth of rice compared with non-treated control. The greatest effect was observed from seed-coating treatments of 100 mg/L MeSA (Fig. 3). On average, seed priming treatments significantly increased the shoot length by 40.78% (*F*_4,30_ = 14.88; *P* < 0.0001), 24.31% (*F*_4,30_ = 17.05; *P* < 0.0001), 20.90%(*F*_4,30_ = 10.73; *P* < 0.0001), 35.34% (*F*_4,30_ = 7.30; *P* < 0.0001), 25.90% (*F*_4,30_ = 7.88; *P* < 0.0001) and 34.82% (*F*_4,30_ = 11.10; *P* < 0.001) for IR 20, IR 50, IR 64, ASD 16, ASD 19 and ADT 46 respectively compared with control. The same trend was also noticed in root length after treatment with 100 mg/L MeSA. For example 54.46% increased root length was observed in ASD 16 after treatment with 100 mg/L MeSA which was significantly different from control (*F*_4, 30_ = 11.25; *P* < 0.0001). Insignificant difference was observed between treatments of 75 and 100 mg/L MeSA.

**Figure 4:**
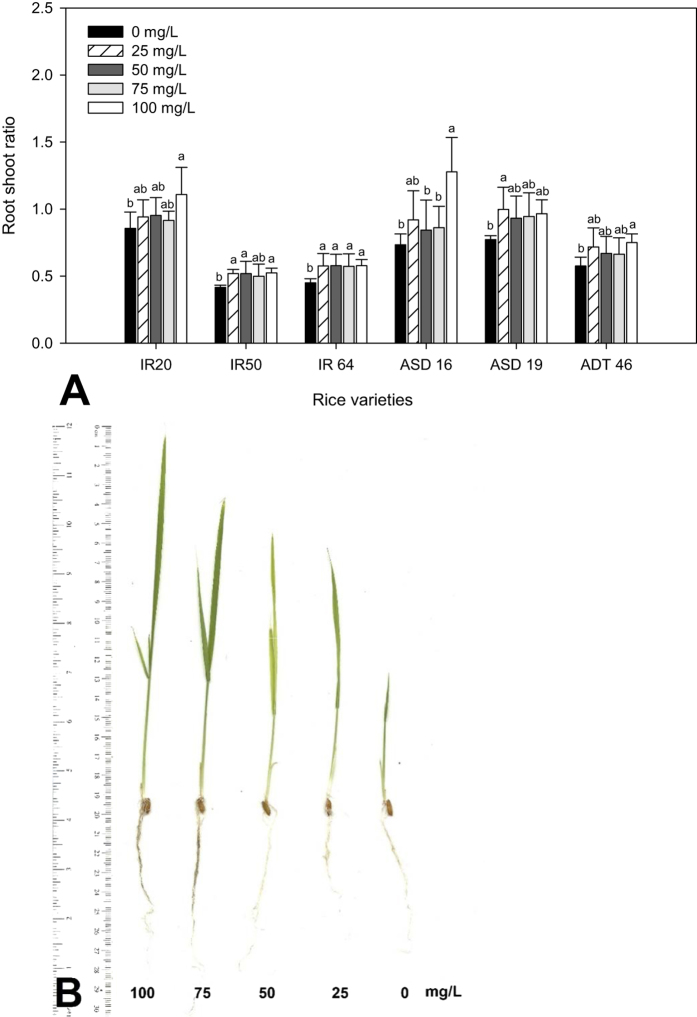
Root and shoot ratio of rice varieties (**A**) (A-IR 20; B-IR 50; C- IR 64; D-ASD 16; E-ASD 19; F-ADT 46) after treatment with MeSA. Mean (±SEM) followed by the same letter in an individual experiment indicate no significant difference (P < 0.05) in a Tukey’s test (Treatment concentration in mg/L). (**B**) Root and shoot growth after treatment with MeSA of ASD 16 rice cultivar.

Treatment of seeds with MeSA also improved root: shoot ratio of the rice seedlings under greenhouse condition. For ‘ASD 16’, the effect of MeSA seed treatment was significant for root: shoot ratio (*F*_4,30_ = 4.44; *P* < 0.006) (Fig. 4A). Interestingly, there was a non-linear response for IR 50 and IR 64, root: shoot ratios and they were not significant between 25–100 mg/L treatments (*F*_4, 30_ = 3.73; *P* < 1.00 and *F*_4, 30_ = 3.98; *P* < 1.00 for IR 50 and IR 64 respectively) (Fig. 4A). Further we have observed significant growth of root and shoot morphologically after seed were treated with MeSA (Fig. 4B).

**Figure 5:**
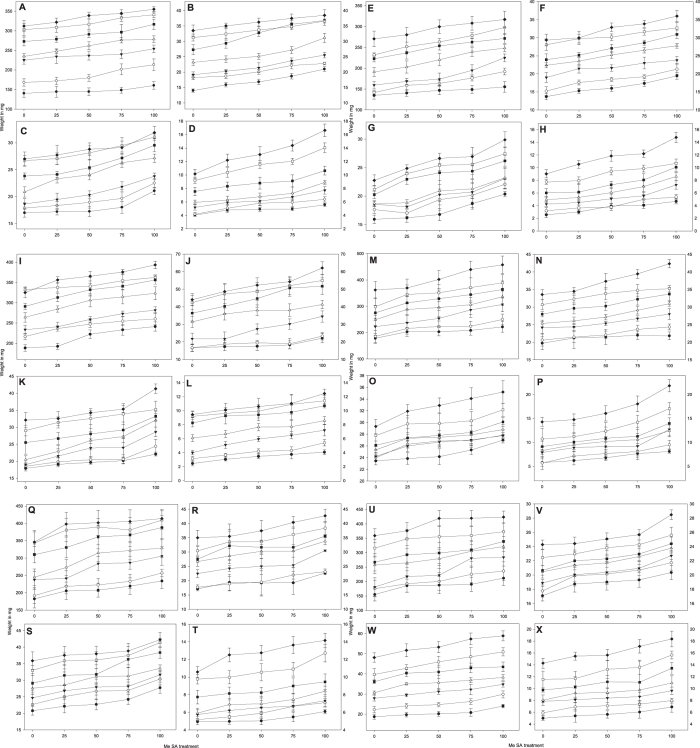
Fresh (**A**,**E**,**I**,**M**,**Q**,**U**) and dry (**B**,**F**,**J**,**N**,**R**,**V**) weight (mg) of shoot; fresh (**C**,**G**,**K**,**O**,**S**,**W**) and dry (**D**,**H**,**L**,**P**,**T**,**X**) weight (mg) of root of IR 20,IR 50, IR 64, ASD 16, ASD 19 and ADT 46 rice varieties after treatment with MeSA in greenhouse condition (-●-10^th^ day -○- 12^th^ day -▼- 14^th^ day-△- 16^th^ day -□- 18^th^ day-■- 20^th^ day -♦-22^nd^ day) (MeSA treatment doses are mg/L).

All the seed treatments had significant effects on fresh and dry weight of shoot and root (Fig. 5). Shoot fresh weight significantly increased in seedlings raised from seeds treated with 75 and 100 mg/L MeSA. Maximum shoot fresh weight of 457.4 mg was attained in ASD 16 seeds treated with 100 mg/L MeSA after 20 days as compare to control (361.8 mg) (Fig. 5). However minimum shoot fresh weight of 270.66 mg was observed in control seeds of IR 50 after 20 days. Shoot and root fresh and dry weights of ‘IR 20, IR 50, IR 64, ASD 16, ASD 19 and ADT 46 treated and untreated plants are shown in Fig. 5A–X. Seed treatment with 100 mg/L MeSA significantly increased shoot fresh weights (*F*_4,20_ = 14.69, *P* < 0.0001-IR 20; *F*_4,20_ = 5.75, *P* < 0.003-IR 50; *F*_4,20_ = 7.69, *P* < 0.001-IR 64; *F*_4,20_ = 7.18, *P* < 0.001-ASD 16; *F*_4,20_ = 3.22, *P* < 0.035-ASD 19; *F*_4,20_ = 3.91, *P* < 0.017-ADT 46) and dry weights (*F*_4,20_ = 24.08, *P* < 0.0001-IR 20; *F*_4,20_ = 69.90, *P* < 0.0001-IR 50; *F*_4,20_ = 7.88, *P* < 0.001-IR 64; *F*_4,20_ = 29.06, *P* < 0.001-ASD 16; *F*_4,20_ = 7.60, *P* < 0.001-ASD 19; *F*_4,20_ = 13.31, *P* < 0.0001-ADT 46) at 22 days post treatment. The same linear growth pattern was observed both in fresh and dry weight of root after seed treatment with MeSA. In general, a positive relationship was observed between seed treatment concentration rate and root and shoot weights (Fig. 5).

### Control of bacterial blight under greenhouse condition

**Figure 6:**
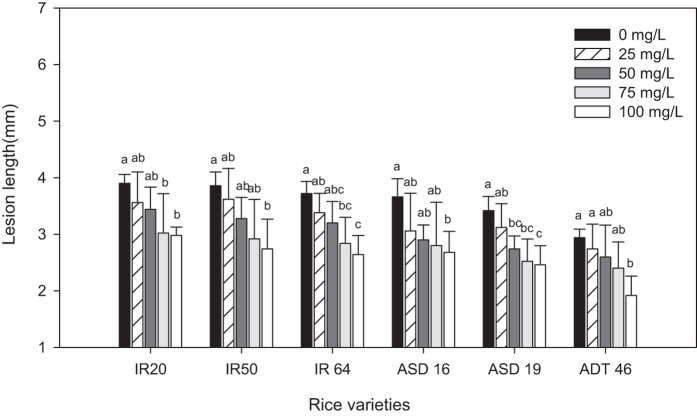
Mean lesion length (mm) caused by *X. oryzae* after treatment with MeSA. Means followed by the same letter were not significantly different (P < 0.05) according to a Tukey test) (Treatment concentration in mg/L).

**Figure 7:**
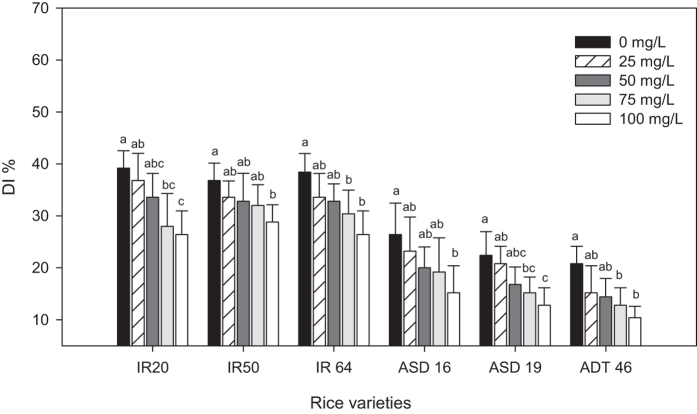
Disease index (DI in percentage) of rice after treatment with MeSA. Means followed by the same letter were not significantly different (P < 0.05) according to a Tukey test) (Treatment concentration in mg/L) Fig. 1.

The effect of MeSA seed treatment in suppressing bacterial blight disease was analyzed under greenhouse condition. Bacterial blight disease lesion length (mm) (Fig. 6) and Disease index (Fig. 7) was found to be significantly lower in 100 mg/L MeSA seed treated rice plant. However other lower seed treatments also found to be effective than control. The bacterial blight disease lesion length (mm) was 2.94 mm in control but the ADT 46 rice seed treated with 100 mg/L was inoculated with *X. oryzae* lesion length (mm) measured as 1.92 mm (*F*_4,20_ = 4.39, *P* < 0.01) (Fig. 6). However we did not observe any significant difference between control and 25 mg/L MeSA seed treated rice ADT 46 plant (*F*_4,20_ = 4.39, *P* < 0.93).

The same trend was observed in disease index. The disease index (DI) was higher in (39.2%) control IR 20 plant and the DI was significantly lower in IR 20 rice plant in which the seed was treated with 100 mg/L MeSA (26.40%) (*F*_4,20_ = 6.33, *P* < 0.002) (Fig. 7). Even though three rice varieties (i.e. ASD 16, ASD 19 and ADT 46) are resistant to bacterial blight disease, seed treatment with MeSA further enhance the resistant against the bacterial blight disease and it was proved by the disease index experiment.

## Discussion

Treatment of seeds with MeSA increased rate of germination and rice growth. The amount of effect differed among rice varieties and effects were dose rate dependent. Also, effects appeared to be more pronounced at 75 and 100 mg/L seed treatments under normal greenhouse conditions. MeSA influences seed germination, seedling establishment, cell growth, respiration, stomatal closure, senescence-associated gene expression, basal thermotolerance, nodulation in legumes, and fruit yield^25^,^31^.

Most consistent response to MeSA seed treatment in this research was an increase in rate of emergence of seeds and biomass of the rice plants after sowing. The effect of MeSA was stronger both in ‘IR 20 and IR 50’ than other rice varieties, which produced higher seedling emergence rates (Kalaivani and Senthil-Nathanunpublished data).

This study shows that rice plant could be induced by exogenous application of the signaling compounds MeSA. The allelopathic potential of the root and shoot of rice thus increased after treatment with MeSA (Figs 1, 2, 3, 4, 5). The same effect was observed in the rice strains IAC165 and Huajingxian^32^. In the present study MeSA treatment with 100 mg/L of rice seed showed enhanced effects on emergence and biomass of rice seedlings. Other studies have also shown that natural plant products can enhance defense system of plant along with protection from pest and diseases^33^,^34^,^35^. Further plant hormonal and other treatments enhanced seedling vigor as measured in longer shoot and root lengths, with increased fresh and dry weights. Accelerated cell division and enlargement of cell explain the production of vigorous seedlings by pre-sowing seed treatment with plant growth regulators^16^.

Several studies have shown impact of MeSA seed treatment on germination and early seedling growth. Enhanced germination, emergence and growth were recorded in wheat, *Triticum aestivum* L. when the seeds were subjected to pre-sowing seed-soaking treatment in salicylic acid^36^. 3 Senthil-Nathan *et al*.^37^ reported that neem treatment on rice resulted in increased production and diseases tolerance than control. Hayat *et al*.^38^ observed the grains of wheat (*Triticum aestivum* L. cv. Raj-3077) were soaked in 0, 10^−5^, 10^−4^ and 10^−3 ^M aqueous solutions of seedlings possessed significantly greater leaf numbers, fresh and dry mass. The present finding gained support from these previous reports.

Shakirova *et al*.^39^ conclude that pre-sowing *T. aestivum* seed treatment with SA leads to an activation of germination and seedling growth. The enhancement results in the division of root apical cells which is an important contribution to the growth stimulating effect of SA. Shakirova^36^ has confirmed with field experiments. In the field, wheat plants pretreated with SA were characterized with increased size of ears, grain yield, indicating prolonged effect of pre-sowing treatment of seeds, which produced stimultative effect on the productivity of wheat, at harvest.

Rate of seedling emergence was increased approximately 25% at seed treatment with 75 and 100 mg/L MeSA. Emergence of ‘IR 20, IR 50 and ASD 16’ seedlings was significantly faster in MeSA treated seeds, but it is not linear with treatment doses. The percentage of emergence (20, 40, 60 and 80%) at the different seed treatment rates indicated that emergence increased with seed treatment concentration rate, with faster emergence from seeds treated with 100 mg/L MeSA. The increased percentage of emergence and biomass with increasing dose is probably due to increased SA in plant cells, as reported in other crops. For example, increased percentage of emergence and growth was observed by Singh and Usha^40^ and Dolatabadian *et al*.^41^ in wheat seeds, Tavares *et al*.^28^ in rice, Khodary^42^ in maize, Jadhav and Bhamburdekar^43^ and Szepesi *et al*.^44^ in tomato, Martin-Mex *et al*.^45^ in African violet, McCue *et al*.^46^ in pea seeds, Almeida *et al*.^47^ in wheat and barley seeds which revealed that MeSA accelerates germination greatly. Seed treatment with MeSA may help in the metabolic pentose phosphate pathway, benefiting the hydrolysis of reserves and increasing the availability of energy to the germination process and seedling emergence^25^.

Several reports were published in the last decade demonstrating the role of SA applied as seed soaking treatment on various physiological processes^44^,^45^,^46^,^47^. It was shown that the pre-soaking of pea seeds in SA had a beneficial effect on growth and photosynthesis^48^.

MeSA treatments produced measurable effects on root and shoot growth of rice seedling varieties. Gutierrez-Coronado *et al*.^49^ reported that foliar application of SA to the shoots of soybean plants positively affected the size of the root. Furthermore Sandoval-Yepiz^50^ reported similar findings with *Tagetus erecta*, using low concentrations (10^−10^ M) of SA. Increased shoot growth was observed in *Clitoria* sp. after application of salicylates^51^. A more in-depth study would be required to determine the specific modes of action of MeSA on non-linear growth of roots and shoots in different crops.

The increase observed in shoot and root weight when treated with MeSA, was dose dependent (75 and 100 mg/L). This response suggests that a greater portion of photosynthates was distributed to the roots than the shoots in seedlings developing from nontreated seeds as compared with seedlings developing from MeSA treated seeds^25^,^36^,^52^. The promoting effect of SA on the leaf area was attributed to its important roles on activating cell division, biosynthesis of organic foods, water uptake and stomatal resistance and tolerating water deficit better. In addition, mentioned that improving effect of SA on the ready availability of nutrients (i.e. N, P, K, Ca, and Mg) to the plant. Increased levels of mineral after treatment with SA were observed in maize^53^, tomato^44^ and cucumber^54^. These results are in support with many other researchers who reported an increased amount of nutrients in roots and shoot after treatments with SA^27^,^55^,^56^.

According to Stout *et al*.^57^ and Vazirimehr *et al*.^27^ plant seeds treated with SA have higher levels of amino acids, plant growth, ion uptake, transport enzyme activity and synthesis of plant hormones, that increase the plant responses to these proteins and these events provide significant increases in production and reduce time for the establishment of the crop field, being more tolerant against abiotic stresses. The data of the present study support the above hypothesis.

For more than two decades the plant hormone salicylic acid (SA) and its derivatives (MeSA) has been studied for its use to protect the plant. However, its systemic acquired signaling act in plants, particularly in defense against plant disease causing microbes, has only become more interest during last two decades^31^,^57^. Our research has clearly pointed out the MeSA has a potential to control the bacterial blight disease. Also seed treated with 100 mg/L has less blight lesion and diseases index. It was evident that SA and its derivatives act as a critical defense signal has come from analyses of plants in which endogenous SA levels were triggered or altered. It was proved in series of experiments in Arabidopsis (Transgenic tobacoo) expressing the bacterial *nahG* gene, encoding the SA-metabolizing enzyme salicylate hydroxylase^31^,^58^,^59^. In addition, endogenous SA levels increase in barley resisting infection by the bacterial pathogen *P. syringae* pv. *syringae*^60^. Also in rice homolog enhances resistance to the bacterial blight-causing *X. oryzae*^61^,^62^,^63^.

MeSA seed treatments (75 and 100 mg/L) significantly increased the emergence and early growth of rice plants. But the effect was non-linear across treatment concentrations and rice varieties. Further we conclude that use of MeSA in seed treatment has great potential for increasing uniform emergence, root and shoot length, root and shoot biomass. Thus, treatment of seeds with SA may produce favorable effects on rice growth. Also MeSA seed treatment has better protection against blight diseases.

## Methods

### Plant material and seed treatments

A commercial formulation of methyl salicylate (Methyl salicylate, Sigma-Aldrich) was used for all experiments. The rice varieties ‘IR 20, IR 50, IR 64, ASD 16, ASD 19 and ADT 46’ were used for this study. ‘IR 20, IR 50, IR 64’ are susceptible, conventional, semi-dwarf, long-grain aromatic variety with relatively high seedling vigor, whereas ‘ASD 16, ASD 19 and ADT 46’ are moderate resistance, conventional, semi-dwarf long grain variety with low seedling vigor. Rice were surface disinfected by immersing in 2% sodium hypochlorite for about two minutes and washed several times with sterilized water, then dried in sterilized filter paper.

Salicylate formulations and treatments consisted of increasing levels of 0, 25, 50, 75 and 100, mg/L of MeSA. A stock solution of MeSA 100 mg/L was used for successive dilutions in distilled water. The seeds were treated by applying 2 ml/kg of seed in each respective MeSA concentration according to methodology described by Tavares *et al*.^28^. The solution of MeSA with the different doses was directly placed at the bottom of the zip lock plastic bag. Then 250 g of seeds were added into each bag. They were agitated for 10 minutes. The seeds were spread out on clean paper and left to dry at room temperature for 24 hours. Treated seeds were used for further experiments.

### Seed treatments under greenhouse conditions

‘IR 20, IR 50, IR 64, ASD 16, ASD 19 and ADT 46’ rice plants were grown by sowing five seeds of a single treatment rate (0, 25, 50, 75 and 100 mg/L) in 0.5 L pots in a greenhouse. There were 7 pots (replicates) per treatment. The potting soil was composed of two parts of autoclaved silt loam soil with one part each of sand and peat moss. The experiment was conducted in early January and, as temperature regulation in the greenhouse was poor, temperatures fluctuated from 22° to 32 °C. Lighting was ambient, and pots were watered as needed.

Pots were observed daily for emergence of hypocotyls above the surface of the soil after sowing. The experiment was terminated after 12 days. For statistical analysis, days until 20%, 40% and 60% seedling emergence were calculated for each pot. At 12^th^ day after sowing, all seedlings were removed from pots, counted, and cleaned with water. Maximum root length (cm/plant) shoot length (cm/plant), weight (both wet and dry weight in mg) for each plant were determined by using a ruler and monopan balance.

Germination was scored daily by counting emerging plant shoots. The experiment was terminated after 22 days. Percent emergence was calculated as in the greenhouse experiment. Roots and shoots were weighed after cleaning and patting dried with a paper towel. After taking fresh weights, plants were dried in an oven at 40 °C for two days and weighed.

### Effect of MeSA seed treatment on the bacterial blight disease of rice under greenhouse conditions

Rice seed treated with MeSA were sown in earthen pots. The potting soil was composed of two parts of autoclaved silt loam soil with one part each of sand and peat moss and maintained under greenhouse conditions. Five replicates of five pots, each containing 5 plants, were maintained. *X. oryzae* inoculum was sub-cultured and grown individually in nutrient broth for 48 h and then centrifuged for 8000 × *g* for 15 m and then resuspended in sterile distilled water at 2 × 10^7 ^CFU/ml. Inoculation was done on twenty-day old plant leaves by scissors-dip method^30^. In each pot, the number of plants showing symptoms was counted and lesion length was measured at 14 days post-inoculation. The values are represented as percent disease incidence (DI) (Formula 1) and mean lesion length (MLL) (Formula 2).


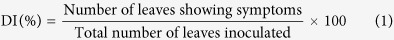


mean variance of disease index and disease incidence of five replications containing five plants in each pot were determined by Tukey-family error test (P < 0.05) by using Minitab^®^17


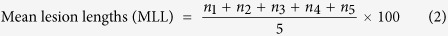


### Statistical analysis

Treatment effects on the percentage of emergence, root and shoot weight, and root: shoot ratio, in rice plants were analyzed by analysis of variance (one-way ANOVA), and the treatment means were compared by Tukey-family error test (P < 0.05) by using Minitab^®^17 software package. The data on above said experiments were arcsine transformed (except percentage of emergence) before undergoing statistical analysis.

## Additional Information

**How to cite this article**: Kalaivani, K. *et al*. Effect of methyl salicylate (MeSA), an elicitor on growth, physiology and pathology of resistant and susceptible rice varieties. *Sci. Rep.* **6**, 34498; doi: 10.1038/srep34498 (2016).
